# Ribosomally Synthesized and Post-Translationally Modified Peptides Assembled by ThiF-like Adenylyltransferases: Recent Advances and Future Perspectives

**DOI:** 10.3390/molecules30132821

**Published:** 2025-06-30

**Authors:** Shaozhou Zhu, Yan Liu, Hang Wang, Jiabei Sun, Jing Yao, Haiwei Huang

**Affiliations:** 1 National Institutes for Food and Drug Control, Beijing 102629, China; 2 NMPA Key Laboratory for Quality Research and Evaluation of Chemical Drugs, Beijing 102629, China

**Keywords:** ThiF-like adenylyltransferase, tailoring enzymes, natural products, biocatalysis, post-translational modification, synthetic biology

## Abstract

Advances in whole genome sequencing have transformed GenBank into a veritable goldmine of uncharacterized and predicted proteins, many of which still await functional characterization. Notably, natural product biosynthetic pathways are often organized in gene clusters, unlocking thrilling avenues for the discovery of novel metabolites and distinctive enzymatic reactions. In this review, we focus on the versatile ThiF-like adenylyltransferase superfamily (TLATs), a group of enzymes essential for the biosynthesis of a diverse array of ribosomally synthesized and post-translationally modified peptides (RiPPs). Recent researches have revealed that TLATs are widespread in numerous yet uncharacterized RiPP biosynthetic pathways, highlighting significant gaps in our understanding of their extensive catalytic potential. Here, we critically review the latest insights into RiPP gene clusters containing these enzymes, discussing the natural products they generate, their enzymatic functions, catalytic mechanisms, and promising directions for future research.

## 1. Introduction

The rapid advancement of high-throughput genome sequencing has significantly expanded the protein sequence repository in GenBank, with over 5.1 billion sequences recorded in its 2024 release (version 264.0) [1]. Yet, the majority of these genes remain functionally uncharacterized, waiting for further exploration [1]. To manage this vast dataset, GenBank employs algorithms such as hidden Markov models (HMMs) to group uncharacterized proteins into superfamilies based on amino acid sequence similarity and conserved domains [2]. Proteins within the same superfamily typically share similar catalytic mechanisms or structurally similar domains, thus providing a convenient basis for automated genome annotation and offering initial predictions about their physiological roles [3].

Despite these efforts, precisely defining the biological functions of most proteins in GenBank remains a significant challenge [1]. Notably, the phenomenon of gene clustering offers a considerable advantage in clarifying the functions of enzymes involved in microbial natural product biosynthesis [4,5]. Moreover, this phenomenon streamlines the discovery of novel natural products and the characterization of enzyme functions by making the resulting compounds readily accessible for analysis [4,5,6]. Given these considerations, genome mining has emerged as an effective approach for discovering novel microbial natural products [7,8,9]. Among these products, RiPPs are particularly well suited for genome mining owing to their unique biosynthetic pathways [10]. Unlike non-ribosomal peptides (NRPs), which depend on large, multi-modular enzyme complexes to incorporate non-proteinogenic amino acids, RiPPs achieve comparable chemical diversity through post-translational modifications (PTMs) of ribosomally synthesized precursor peptides [10,11,12]. This process endows RiPPs with diverse structural features and bioactivities, including antifungal, antibacterial, allelopathic, and antiviral properties [10].

Generally, RiPPs can be further divided into diverse subfamilies, such as lasso peptides and lanthipeptides, based on their biosynthetic mechanisms and structural traits [10,13,14,15]. Despite their diversity, RiPP biosynthesis follows a conserved framework. The process typically begins with the ribosomal synthesis of a long precursor peptide composed of an N-terminal leader sequence and a C-terminal core region designated for PTMs [10,11]. Tailoring enzymes then recognize the leader sequence and sequentially install various PTMs into the core region. Finally, peptidases remove the leader sequence—sometimes accompanied by N-to-C cyclization—to yield the mature bioactive product. This consistent mechanism greatly enhances the efficiency of genome mining for the discovery and analysis of RiPPs [8].

Recent advances have introduced a variety of genome mining strategies for identifying RiPPs, including approaches that focus on the precursor peptide, the modification enzymes, or the regulatory elements [8,16]. For instance, RiPPs modified by radical S-adenosylmethionine (RaS) enzymes showcase the efficacy of these approaches [17]. Recently, Seyedsayamdost and colleagues used a strategy targeting quorum-sensing regulatory operons and, through sequence similarity network analysis, identified approximately 600 RaS-RiPP biosynthetic gene clusters [17]. These clusters were sorted into 16 subfamilies based on the similarity of their precursor peptides [17]. Further in vitro studies have uncovered nearly a dozen novel and diverse peptide cross-linking reactions, all catalyzed by RaS enzymes, significantly broadening our understanding of the transformations these enzymes can mediate [17,18,19,20]. Tong et al. recently identified a new class of mini lanthipeptide synthetases and a new family of RiPPs of the enterofaecin type using a similar strategy that focuses on regulatory genes [21,22]. These discoveries highlight the value of this approach in uncovering new RiPP families, as it can elucidate biosynthetic pathways that traditional homology-based methods struggle to characterize due to significant sequence divergence. Furthermore, the enzymes responsible for modifications in these complex biosynthetic pathways frequently catalyze intricate transformations, revealing novel or unforeseen reaction mechanisms [17,19,22]. Here, we focus on the ThiF-like adenylyltransferase superfamily as a representative case that illustrates the challenges, achievements, and future directions in studying uncharacterized biosynthetic proteins in this dynamic field.

ThiF-like adenylyltransferases constitute an evolutionarily conserved protein family that plays pivotal roles in both primary metabolism and secondary metabolic pathways. In *Escherichia coli*, ThiF is a key enzyme in thiamine (vitamin B1) biosynthesis, specifically in forming the thiazole moiety [23,24]. Acting as an E1-like adenylyltransferase, ThiF uses ATP to adenylate the C-terminal glycine of the sulfur carrier protein ThiS, forming a high-energy acyl-adenylate intermediate [24,25]. This is followed by the creation of a thioester bond between ThiS and a conserved cysteine on ThiF, priming ThiS for sulfur incorporation to produce ThiS-thiocarboxylate, which transfers sulfur to the thiazole precursor [25]. Structurally, ThiF resembles eukaryotic ubiquitin-activating enzymes and the molybdopterin biosynthetic protein MoeB, suggesting evolutionary ties among ATP-dependent sulfur transfer systems [26,27]. Recent structural and biochemical studies have illuminated ThiF’s catalytic cycle and conformational dynamics, deepening our understanding of its interactions with ThiS and bacterial sulfur mobilization.

**Figure 1:**
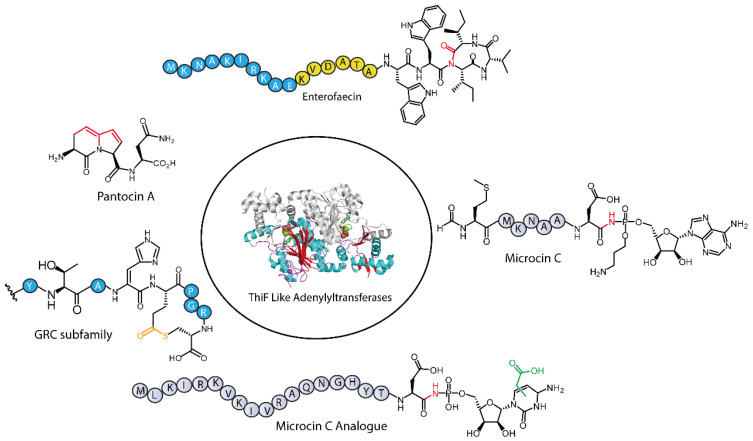
Representative chemical structures of RiPPs synthesized through biosynthetic pathways involving ThiF-like adenylyltransferases.

In the biosynthesis of RiPPs, the function of TLATs is exemplified by studies on the antibiotic microcin C7 (McC). During McC biosynthesis, the enzyme MccB, which features an adenylation domain analogous to those found in ThiF and ubiquitin-like (UBL) protein-activating enzymes, catalyzes the covalent attachment of AMP to the MccA peptide (sequence: fMRT-GNAN) [28,29]. This reaction converts the C-terminal asparagine into an aspartamide, establishing a phosphoramidate bond between its nitrogen and AMP. Recent genome mining efforts have uncovered numerous RiPP biosynthetic pathways involving diverse TLATs, underscoring their significance in natural product biosynthesis (Figure 1) [19,22]. In this review, we will cover recent advances in our understanding of TLATs involved in diverse RiPP biosynthesis from a mechanistic to a structural perspective. We also outline likely future research directions for this protein superfamily.

## 2. TLATs in Microcin C-Type RiPPs Biosynthesis

### 2.1. Functions of Microcin C-Type RiPPs

Microcin C is a peptide-nucleoside antibiotic produced by *E. coli* and other Enterobacteriaceae, which has attracted considerable interest owing to its unique chemical structure and antibacterial mechanism [30,31,32,33]. Initially isolated from the *E. coli* strain BM7006 and first designated as “microcin C7”, it was later independently identified as “microcin C51” [34,35]. Early studies have shown that McC exhibits potent antibacterial activity against a range of Gram-negative bacteria, including *Escherichia*, *Salmonella*, *Shigella*, and *Klebsiella*, with minimum inhibitory concentrations (MICs) in the micromolar range [36]. In addition to effectively suppressing pathogen growth, McC plays a critical role in inducing the persister state in *E. coli* [37]. Recent studies have further shown that McC-like molecules participate in allelopathic interactions among *Synechococcus* species, suggesting that these natural products may have diverse physiological functions [38]. Moreover, in vivo studies in animal models indicate that McC has strong probiotic activity in the gastrointestinal tract [39]. For example, in a mouse model, secretion of McC by *E. coli* strain H22 significantly inhibits pathogen proliferation, highlighting its potential for infection control [39]. In chicken models, Microcin C7 not only markedly increases the population of *Lactobacilli* in the cecum but also decreases the overall bacterial load and the numbers of *E. coli*, thus modulating the gut microbiome to promote enhanced growth performance [40]. Given its unique mechanism of action and efficacy against multidrug-resistant strains, McC is increasingly viewed as a promising candidate for novel antibacterial drug development. Advances in elucidating its chemical structure and mode of action have laid a robust foundation for its further pharmaceutical exploration [41,42,43].

### 2.2. Molecular Structure and Biosynthetic Pathway of Microcin C-Class Natural Products

McC derived from *E. coli* exhibits a unique “Trojan horse” mechanism that strategically integrates a peptide carrier module with a core toxic moiety consisting of a nonhydrolyzable amino acid-adenosine conjugate [44]. Its core structure consists of a heptapeptide covalently linked to adenosine monophosphate (AMP) through an N-acyl phosphoramidate bond. Notably, the N-terminal methionine of the heptapeptide is formylated, while the phosphate group of AMP is further esterified with a propylamine group. These modifications significantly enhance the binding affinity for the target enzyme, aspartyl-tRNA synthetase (AspRS), thereby markedly increasing its antibacterial potency [45]. The heptapeptide serves a dual role: it acts as the precursor peptide and functions as a “transporter module” to facilitate cellular uptake through the host YejABEF transport system [46,47]. Additionally, unmodified heptapeptide fragments have been reported to exhibit intrinsic antibacterial activity, offering alternative perspectives on the antibacterial mechanism of these compounds [48]. Upon entering the cell, the peptide undergoes sequential degradation: a deformylase first removes the formyl group, followed by progressive cleavage from the N-terminus to the C-terminus by aminopeptidases PepA, PepB, and PepN, ultimately producing a propylamino-modified isoaspartyl-adenosine [49]. This product mimics aspartyl-adenosine, thereby inhibiting AspRS activity. The inhibition leads to the accumulation of uncharged tRNA^Asp^, triggering translation arrest and the stringent response, which ultimately culminates in growth termination [32,45].

**Figure 2:**
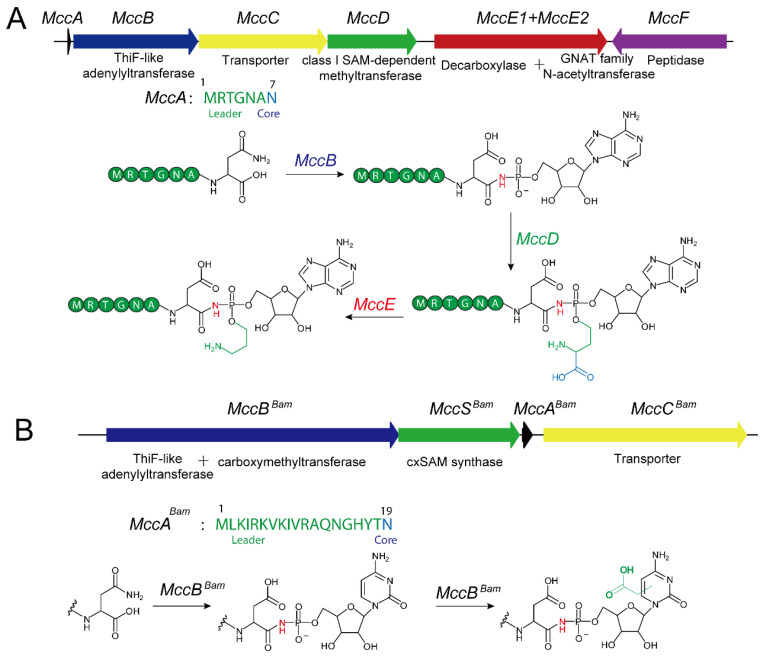
(**A**) Biosynthetic gene cluster for Microcin C from *E. coli* and proposed biosynthetic pathway; (**B**) Biosynthetic gene cluster for Microcin C analog from *B. amyloliquefaciens* DSM7 and proposed biosynthetic pathway.

The biosynthesis of McC-type natural products follows the typical RiPP biosynthetic pathway [10]. In *E. coli*, the McC biosynthetic gene cluster has been fully characterized and comprises six genes (Figure 2) [50]. The MccA gene encodes a precursor peptide of seven amino acids (sequence: MRTGNAN). Adenylyltransferase MccB, in the presence of ATP and Mg^2+^, catalyzes the covalent attachment of a nucleotide to the precursor peptide, while MccD and MccE further modify the product by installing a phosphoramidate propyl moiety [51,52]. These modifications not only enhance the cytotoxicity of the peptide-nucleoside but also enable it to evade recognition by host immunity proteins. It is noteworthy that although most *Salmonella* strains exhibit tolerance toward the peptide-nucleoside conjugates, they are more sensitive to the propyl-modified compounds [53]. The introduction of the propyl group occurs via a two-step reaction: in the first step, the class I methyltransferase MccD transfers the 3-carboxy-3-aminopropyl moiety from S-adenosylmethionine (SAM) to the peptide-nucleoside substrate, concomitantly releasing methylthioadenosine (MTA) [52]. In the second step, the N-terminal decarboxylase domain of MccE catalyzes the decarboxylation of the 3-carboxy-3-aminopropyl-modified intermediate [52].

In contrast, the biosynthetic mechanism of Microcin C analogs from *Bacillus amyloliquefaciens* displays marked structural and biosynthetic differences (Figure 2) [54]. Their gene clusters encompass only four key genes and yield a cytidine antibiotic bearing a carboxymethyl modification [54]. Here, the MccA^bac^ gene encodes an elongated precursor peptide of 19 amino acids. Unlike in *E. coli*, the corresponding adenylyltransferase (MccB^bac^) in *B. amyloliquefaciens* attaches a cytidine monophosphate to the precursor peptide’s C-terminus, and the cytosine moiety subsequently undergoes an additional carboxymethylation catalyzed jointly by the C-terminal domain of MccB and an MccS-like enzyme acting as the carboxymethyl donor [54]. Studies indicate that these cytidine-bearing analogs are bioactive and target AspRS in a manner similar to their *E. coli* counterparts [54].

Beyond the genes directly involved in McC biosynthesis, the gene clusters frequently harbor genes associated with self-immunity. Because McC producers are susceptible to the accumulation of isoaspartyl-nucleoside intracellularly, immunity genes are typically embedded within the McC gene cluster [31,55]. For example, the C-terminal domain of the GNAT (Gcn5-related N-acetyltransferase) of MccE mediates acetylation at the α-amine of the processed McC, thereby providing immunity by blocking their interaction with cognate aminoacyl-tRNA synthetases [56]. Additionally, the MccF protein encoded within the *E. coli* McC cluster mediates self-immunity by cleaving the terminal aspartate residue or oligopeptide from processed or mature McC molecules [57,58]. Remarkably, homologs of MccF are not only present in multiple McC gene clusters but also distributed among independent bacterial genomes. Recent studies have also shown that histidine-triad (HIT) hydrolases associated with the biosynthetic gene clusters of certain microcin C homologs confer resistance to McC-like compounds by cleaving the phosphoramidate bond of the toxic aspartamide-adenylate intermediate, thereby neutralizing its activity [53].

### 2.3. Structure and Catalytic Mechanism of TLATs in Microcin C-Type RiPPs Biosynthesis

MccB is the central enzyme in McC biosynthesis, whose primary function is to append AMP to the C-terminal asparagine (Asn) of the MccA peptide, thereby converting it into an isoaspartyl amide and forming a chemically stable N-P bond [28,29]. In vitro studies by Walsh and colleagues have demonstrated that this catalytic process consumes two molecules of ATP and proceeds via a succinimide intermediate through a two-step mechanism [59]. In the initial step, MccB uses one molecule of ATP to activate the C-terminal Asn of MccA, forming an acyl-adenylate intermediate; this is followed by an intramolecular rearrangement with expulsion of AMP to yield a succinimide intermediate [59]. During the second step, the nitrogen of the succinimidyl moiety attacks the α-phosphate of a second ATP molecule, thereby opening the succinimide ring and converting the C-terminal asparagine to isoaspartate, ultimately generating the bioactive adenylated product. Unlike traditional adenylylation reactions, MccB does not form thioester intermediates and specifically modifies the C-terminal Asn, reflecting its unique catalytic features [59].

**Figure 3:**
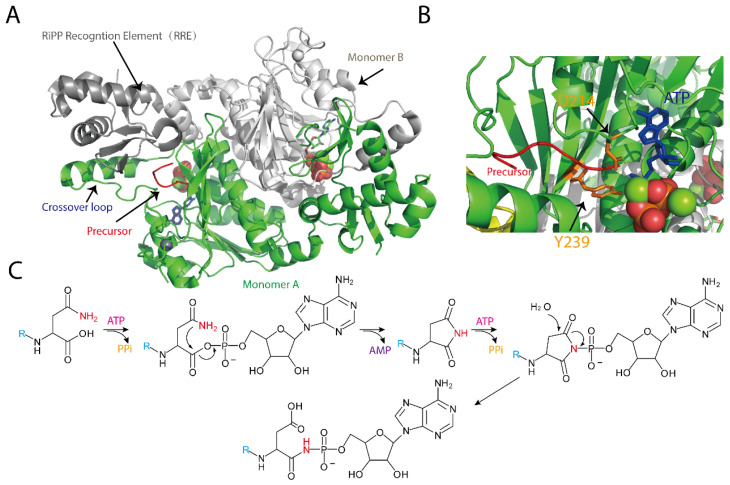
(**A**) Structure of MccB homodimer bound to precursor peptide. One monomer is colored in gray and the other in green. The RiPP recognition element and the crossover loop are marked; (**B**) The active site of the adenylation domain binding with the precursor (key residues required for catalysis is shown); (**C**) The mechanism of P-N bond formation by MccB using two molecular ATP.

Crystallographic studies of MccB have further elucidated its functional basis (Figure 3) [28,29]. The enzyme exists as a homodimer, with each monomer comprising two key regions: a ~260-residue adenylylation domain that is analogous to UBL-activating enzymes and orchestrates the two-step adenylylation reaction, and a “peptide clamp” structure formed by approximately 90 N-terminal residues (the RiPP recognition element, RRE domain) from one monomer in conjunction with 20 C-terminal residues and a central “cross-over loop” from the other monomer, which together secure the MccA peptide for precise substrate engagement [28,29]. Structural analysis of the MccB-ligand complex reveals that the peptide-binding domain exhibits considerable flexibility and may undergo significant conformational rearrangements upon substrate binding, thereby positioning ATP in proximity to the activated MccA for efficient nucleophilic attack (Figure 3) [28,29]. These structural features not only underpin the high catalytic efficiency of MccB but also provide a molecular basis for its strict substrate specificity.

With respect to substrate specificity, MccB exhibits a remarkable feature: in vitro assays demonstrate its ability to conjugate the precursor peptide MccA with a spectrum of nucleotides (AMP, CMP, GMP, or UMP), underscoring a surprisingly permissive specificity for diverse nucleoside triphosphate (NTP) substrates. However, in vivo, the reaction is highly specific, producing exclusively the AMP-linked peptide-nucleoside conjugate [54]. Moreover, MccB imposes stringent sequence requirements on MccA. Systematic mutagenesis has shown that substitutions at Thr-3 and Asn-7 of MccA dramatically impair MccB activity, whereas other positions are more permissive [60]. This selectivity is closely coupled with the configuration of the active site and the peptide clamp structure. Notably, the N-terminal formylation of the precursor heptapeptide is critical for efficient catalysis; MccB preferentially processes precursor peptides retaining the formylated methionine (fMet) [29]. Kinetic and binding studies further illustrate that the N-formyl group substantially enhances the binding affinity between MccA and MccB and induces a well-ordered conformation within the crossover loop, thereby optimizing substrate recognition. In addition, the N-formylated peptide exhibits substrate inhibition that cannot be recapitulated by its deformylated counterpart, suggesting a regulatory mechanism to prevent excessive intracellular McC accumulation and mitigate potential toxicity [29].

### 2.4. Genome Mining for Microcin C-Type RiPPs

**Figure 4:**
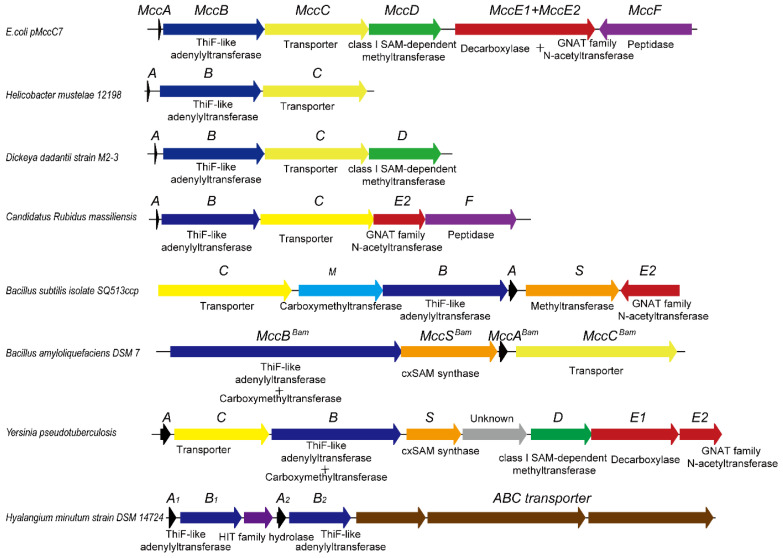
Biosynthetic gene clusters of microcin C-like natural products found across bacterial genomes.

Recent bioinformatic analysis has showed that diverse McC-like gene clusters are widely distributed among bacteria (Figure 4), with several clusters having been validated by in vitro experiments [61]. Intriguingly, McC-type antibiotics can be generally categorized into peptide-nucleoside and peptide-cytidine classes [33,54]. In some cases, gene clusters encoding peptide-nucleoside McC analogs homologous to those in *E. coli* may include just three core genes, MccA, MccB, and MccC, which represent a minimal pathway for peptide-nucleoside biosynthesis. Using MccB as a query, genome mining has revealed that many McC-type gene clusters exhibit complex architectures [33]. Notably, homologs of MccD, MccE, and MccF are arranged in diverse configurations within their respective gene clusters, suggesting that these loci have undergone combinatorial evolution to diversify their biosynthetic pathways.

In gene clusters homologous to the cytidine antibiotic produced by *B. amyloliquefaciens*, most harbor genes associated with carboxymethyl modifications, which are catalyzed by the C-terminal carboxymethyltransferase domain of MccB in conjunction with an MccS homolog serving as the carboxymethyl donor. Interestingly, in the *Bacillus subtilis* isolate SQ513ccp, the carboxymethyltransferase is not fused to the adenylyltransferase, and the gene cluster harbors an acetyltransferase homologous to MccF. In *Yersinia pseudotuberculosis*, the gene cluster includes genes both for the phosphoramidate propyl moiety modification and genes for carboxymethyl modification. Recently, Tsibulskaya et al. demonstrated that an 11-amino-acid-long peptide with a C-terminal modified cytosine residue could be produced by this type gene cluster and could inhibit sensitive cells in the same way as microcin C [62].

Moreover, due to the self-immunity mechanisms intrinsic to McC-producing bacteria, many gene clusters also encode proteins that may function in the degradation of toxic precursor molecules accumulating within the cell, such as AspN-like peptidases or ClpA homologs [33,61]. These findings suggest that, over the course of evolution, bacteria have developed a wide range of antibacterial and self-immunity strategies. A recent example is the gene cluster identified in *Hyalangium minutum* strain DSM 14724, which encodes a novel HIT family hydrolase. In a recent study, Yagmurov et al. demonstrated that hydrolases encoded by these biosynthetic clusters can cleave the phosphoramide bond in the toxic intermediate aspartamide-adenosine, revealing a previously unrecognized mechanism of resistance to McC-like compounds [53]. Looking ahead, in-depth investigations of these novel gene clusters discovered through genome mining, especially those containing previously uncharacterized modification or immunity proteins, are expected to yield new antibiotics with unique chemical structures and reveal novel antibacterial mechanisms, thereby guiding the development of next-generation antibiotics.

## 3. TLATs in Pantocin A-Type RiPPs Biosynthesis

### 3.1. Biological Function of Pantocin A

The genus *Pantoea* maintains a close ecological relationship with plants, comprising a diverse array of species with distinct functional roles. These include plant pathogens that pose significant threats to agricultural production, as well as beneficial strains widely employed in biocontrol [63,64,65,66]. During the interaction, beneficial *Pantoea* strains typically inhibit the growth of plant pathogenic bacteria by synthesizing antimicrobial compounds [67]. For example, *Pantoea agglomerans* Eh1087 could synthesize phenazine type antibiotics, and *Pantoea vagans* C9-1 could generate antibiotics such as Pantocin A, dapdiamide-class herbicolin I [68,69,70,71,72]. Additionally, *P. agglomerans* Eh318 produces both Pantocin A and Pantocin B, the latter exhibiting arginine-reversible activity [73]. The diversity of these natural products markedly enhances the potential of *Pantoea* strains for biocontrol applications [64,65]. Among these compounds, Pantocin A has attracted significant attention due to its unique biological activity and represents a key peptide metabolite isolated from *Pantoea* bacteria [67].

The discovery of Pantocin A emerged from investigations into the antagonistic effects of *Pantoea* strains against *Erwinia amylovora* [73]. In minimal media supplemented with free amino acids, researchers observed that the antimicrobial activity of certain *P. agglomerans* strains was significantly diminished upon the addition of L-histidine, suggesting that its activity is associated with L-histidine biosynthesis [74]. Subsequent isolation and characterization identified this antibiotic as Pantocin A, providing critical insights into the antimicrobial mechanisms of *Pantoea* strains and their biocontrol applications [67]. Further cross-feeding experiments revealed that the tripeptide Ala-Gly-Gly inhibited the suppressive effect of Pantocin A on *E. amylovora*, indicating that Pantocin A enters bacterial cells via a tripeptide transporter [75]. Once inside the cell, Pantocin A disrupts histidine phosphate aminotransferase, a pivotal enzyme involved in L-histidine biosynthesis, by inhibiting the conversion of imidazole acetol phosphate to L-histidinol [75]. This results in an intracellular deficiency of L-histidine. Consequently, Pantocin A demonstrates exceptional efficacy in inhibiting pathogens such as *E. amylovora*, making it a valuable tool for the management of fire blight [63,67,73]. So far, several *Pantoea* strains have been formulated into commercial plant-protection products, such as BloomTime Biological™, BlossomBless™ and BlightBan C9-1™, which are now widely used in Canada, New Zealand and the United States. As alternatives or complements to conventional chemical antibiotics, these products play an essential role in controlling fire blight in apple and pear orchards [76].

### 3.2. Biosynthesis of Pantocin A

**Figure 5:**
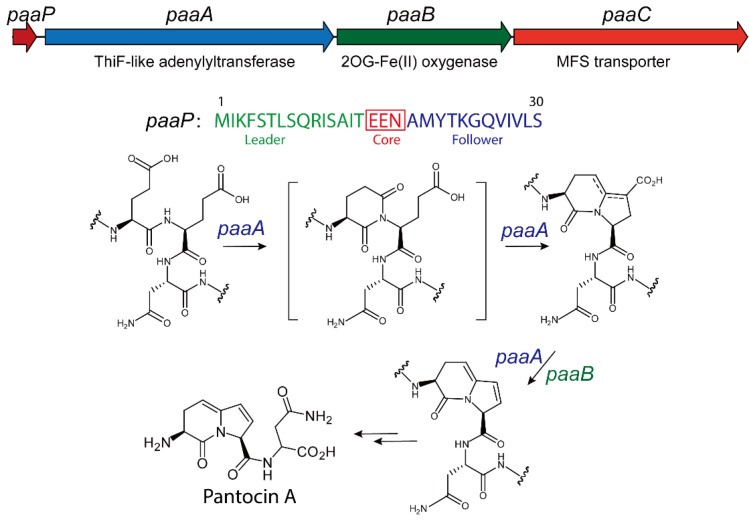
Biosynthetic gene cluster for Pantocin A from *P. agglomerans* and proposed biosynthetic pathway.

Using cosmid library technology, researchers have successfully cloned the biosynthetic gene clusters responsible for Pantocin A production from several antibiotic-producing *Pantoea* strains. The functionality of these gene clusters was validated through heterologous expression in *E. coli*, confirming their ability to direct the biosynthesis of Pantocin A [73]. For example, in *P. agglomerans* Eh318, the Pantocin A biosynthetic gene cluster was identified through cosmid library screening, followed by subcloning to isolate a 3.5 kb DNA fragment encompassing all essential biosynthetic sequences [73]. Sequence analysis disclosed three open reading frames (ORFs)—PaaA, PaaB, and PaaC—alongside a gene, PaaP, encoding a precursor peptide. In *P. vagans* C9-1 and *Pantoea sp.* Eh252, similar gene clusters were discovered with high sequence identify (Figure 5) [67,77,78]. Furthermore, this cluster shows high similarity to those in 23 additional *P. agglomerans* strains. These findings indicate that Pantocin A biosynthesis depends on a conserved gene cluster comprising PaaP, PaaA, PaaB, and PaaC, with core functional sequences remaining highly consistent despite genomic diversity among strains [67].

The biosynthesis of Pantocin A also follows a conserved rule for the RiPP pathway [79]. The PaaP gene encodes a 30-amino-acid precursor which features a fully conserved central region with three critical residues, while its flanking regions display greater sequence variability [80]. The PaaA gene encodes a ThiF-like adenylyltransferase that initiates modification by catalyzing the ATP- and Mg^2+^-dependent dual dehydration and decarboxylation of two glutamic acid residues in the PaaP precursor [80]. On the other hand, the PaaB gene encodes a 2OG-Fe(II) oxygenase family protein presumed to oxidize the precursor via a two-electron dehydrogenation reaction, thereby forming the conjugated alkene characteristic of Pantocin A. The PaaC gene encodes an EamA-family transmembrane transporter that confers resistance to Pantocin A when expressed in *E. coli*, most likely by exporting the antibiotic to protect the host cell [79,80].

To elucidate gene functions, mutation studies were performed in three *Pantoea* strains to assess the impact of specific disruptions on biosynthetic capacity and antibacterial activity. In *P. agglomerans* Eh318, transposon mutagenesis scanning of a 3.5 kb fragment showed that insertions in PaaA, PaaB, or PaaC almost invariably abolished Pantocin A production; intriguingly, a subset of PaaC insertion mutants retained residual antibiotic synthesis but exhibited severely impaired growth [79]. In *P. vagans* C9-1, screening of 300 cosmid-derived mutants identified 26 that lacked Pantocin A production, with all insertion sites mapping to PaaA, PaaB, or the promoter region ~150 bp upstream of PaaP, underscoring the essential roles of both the structural genes and their regulatory elements [71]. Collectively, these data demonstrate that the Pantocin A pathway requires the coordinated function of all four genes, since disruption of any single component compromises product formation [79].

### 3.3. Structure and Catalytic Mechanism of TLATs in Pantocin A Biosynthesis

To elucidate the biosynthetic mechanism of Pantocin A, Ghodge et al. recently characterized PaaA using in vitro assays [80]. In vitro studies have shown that PaaA catalyzes both dual dehydration and decarboxylation in the presence of ATP and MgCl_2_. The data further suggest that PaaB subsequently mediates a dehydrogenation step to generate the conjugated olefin characteristic of Pantocin A [80].

To define the distinct contributions of the N-terminal leader and C-terminal follower peptides during modification, a series of truncated substrates was generated [80]. In vitro assays demonstrated that removal of the final five residues (M1-Q25) or deletion of the entire follower peptide (M1-N18) yielded only the dehydrated product, and addition of the follower fragment (A19-S30) in trans failed to restore further processing [80]. Conversely, substrates lacking the leader peptide—even with an intact follower region—remained unmodified by PaaA, as did constructs bearing shorter leader truncations. These observations establish that the leader peptide (M1-T15) is essential for triggering the initial dehydration, whereas the follower peptide is required for complete maturation, including both dehydration steps and subsequent decarboxylation [80].

Recently, an mRNA display-based assay was developed by Fleming et al. to evaluate enzyme activity by linking peptides to their corresponding encoding RNAs through in vitro ribosomal translation [81]. In brief, N-terminally biotinylated PaaP is displayed and treated with PaaA, followed by GluC digestion to cleave unmodified substrates, and finally enriched via streptavidin affinity purification [81]. A comprehensive single-mutant saturation library (smSVL) was then subjected to 1 µM PaaA for 5, 22.5, and 60 min. Following GluC digestion and streptavidin purification, next-generation sequencing of the recovered pool quantified PaaA’s catalytic efficiency across diverse peptide variants [81]. Interestingly, broad point-mutation tolerance was observed in both leader and follower regions. Of 26 positions, 22 tolerated substitutions, and the follower region proved especially robust, with no single mutation significantly impairing activity. In the leader, however, F4, L7, R10, and I11 substitutions—particularly to aspartate—reduced enrichment, implicating the FXXLXXRI motif in RRE-peptide recognition. The E16 variant remained susceptible to GluC cleavage, whereas E17 was protected, indicating preferential modification at E16. High tolerance at core residue N18 suggests potential for generating novel Pantocin A analogs. Competition fluorescence polarization and isothermal titration calorimetry confirmed the essential role of F4, L7, R10, and I11 in PaaP binding: only the wild-type and T6D peptides effectively competed for PaaA. Mutation of E17 (e.g., E17A) produced a single dehydration product, whereas E16 substitutions—particularly to hydrophobic residues—yielded only partial modification. ^13^C-labeling studies of E17 substrates revealed that PaaA first modifies E16, thereby enabling subsequent cyclization and decarboxylation at E17 [81].

**Figure 6:**
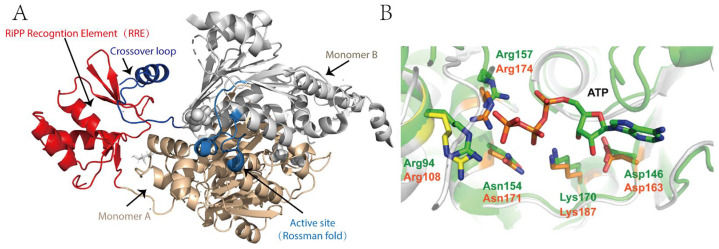
(**A**) Structure of paaA homodimer. One monomer is colored in gray and the other in gold. The RiPP recognition element, the active site, and the crossover loop are marked; (**B**) Overlay of PaaA (orange and yellow, PDB: 5FF5) and MccB ATP complex (green, PDB: 3H5N) ATP-binding residues, showing conserved residues and orientation [80].

Further, X-ray crystallography revealed that PaaA forms a homodimer and adopts a fold closely resembling MccB and the thiazole synthetase TruD (Figure 6) [80]. Each PaaA homodimer comprises two parallel catalytic units, each containing an adenylation domain (residues 100–281 and 321–371) and a smaller globular domain (residues 1–98) homologous to RiPP precursor peptide recognition elements (RREs). The inclusion of an RRE domain distinguishes PaaA and MccB from broader E1-like activating enzymes such as MoeB and ThiF. In the MccB structure, the N-terminal RRE of one subunit engages the crossover loop of the other to form a peptide clamp, and a similar, though more disordered, arrangement is observed in PaaA. These features imply that precursor peptide binding organizes and stabilizes the RRE, accurately positioning the substrate at the active site [80].

The adenylation domain of PaaA aligns closely with that of MccB. As in MccB, each PaaA monomer presents a concave peptide-binding surface: a five-stranded β-sheet that spans the RRE and the Rossmann-fold ATP-binding cleft. Structural superposition with ATP-bound MccB pinpointed key PaaA residues—R174A sharply reduced activity, and K187A abolished substrate conversion. The PaaA active site is substantially larger and more accessible than MccB’s. This expanded pocket likely accommodates the follower peptide and enhances substrate mobility, with an additional shallow groove at its rear further supporting follower-sequence binding [80].

### 3.4. Genome Mining and Synthetic Biology for PantocinA-Type RiPPs

Sequence similarity network analysis reveals that the ThiF family homologous to PaaA is widespread among prokaryotes. A subset of ThiF members contains an RRE domain, indicating their involvement in peptide post-translational modification. BLAST searches using PaaA as the query show that its gene cluster, PaaPABC, is conserved across diverse genomes. Precursor peptides encoded by these clusters, which are homologous to PaaP, often carry substitutions in the core region, with asparagine replaced by lysine, isoleucine, or aspartic acid, thus generating a variety of Pantocin A-like molecules [67,80]. Interestingly, Fleming and colleagues have used an mRNA display-based saturation-mutagenesis library to demonstrate high mutation tolerance at the Pantocin A core residue N18, suggesting that PaaA can be harnessed to synthesize diverse novel analogs [81]. In a more recent study, Voigt and coworkers also found that N18 is highly tolerant of substitution and proposed that a combinatorial synthetic-biology strategy could rapidly produce Pantocin A variants [82].

In future studies, bioinformatics analyses and mutant-library data can guide the chemical synthesis of Pantocin A analogs, which can then be rapidly evaluated for bioactivity to expedite the discovery of antibiotics effective against fire blight. Detailed evaluation of these analogs is expected to further optimize the application of Pantocin A and its derivatives in the precision management of agricultural diseases.

## 4. TLATs in GRC-Type RiPPs Biosynthesis

### 4.1. Biosynthesis of GRC-Type RiPPs

**Figure 7:**
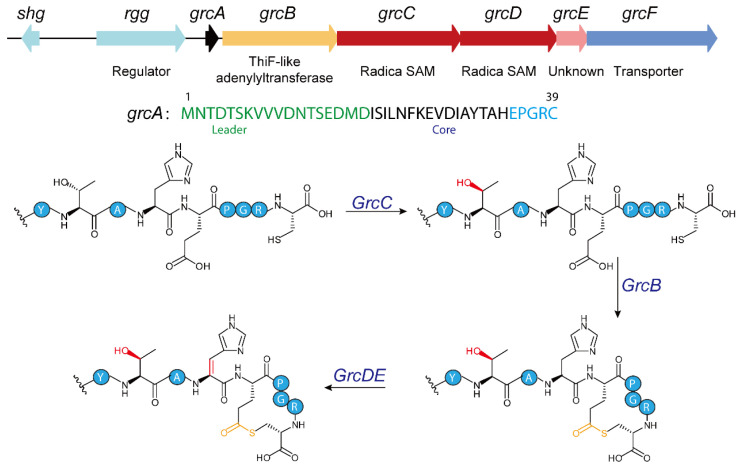
Biosynthetic gene cluster for GRC-type RiPP from *Streptococcus pneumoniae* and proposed biosynthetic pathway.

Recent bioinformatic mining by Seyedsayamdost and colleagues identified roughly 600 RaS-RiPP biosynthetic gene clusters (BGCs) adjacent to quorum-sensing regulatory operons [17,18,19,20,83]. Biochemical characterization has shown that RaS enzymes in these clusters mediate a wide variety of cross-linking reactions [17,18,19,20,84]. Of particular interest is the GRC subfamily (Figure 7), whose distinctive gene architecture comprises a 39-residue precursor peptide (GrcA), a ThiF-like adenylyltransferase (GrcB), two radical S-adenosylmethionine enzymes (GrcC and GrcD), a standalone RiPP recognition element (GrcE), and a transporter (GrcF) [19]. HHpred analysis indicates that GrcB shares homology with the ThiF enzyme of thiamine biosynthesis, suggesting it functions as an adenylyltransferase [17,19].

Expression of GrcA alone in an in vivo heterologous co-expression system produced an unmodified linear precursor peptide by HR-MS and HR-MS/MS. However, co-expression with GrcB yielded a product 18 Da lighter, consistent with dehydration [19]. Further trypsin digestion, HR-MS/MS and NMR spectroscopy confirmed that GrcB catalyzes thiolactone macrocycle formation by cross-linking the C-terminal glutamate (Glu^35^) to cysteine (Cys^39^) [19].

Mechanistic studies, integrating in vivo and in vitro data, indicate that GrcB parallels ThiF’s role in thiamine biosynthesis: it adenylylates the precursor peptide’s C-terminal carboxylate and thereby drives thiolactone formation. In vitro assays confirm that GrcB converts unmodified GrcA into the thiolactone macrocycle only in the presence of Mg^2+^ and ATP [19]. Similarly with other TLATs, GrcB’s function relies on its bimodular architecture, consisting of an N-terminal RiPP recognition element (RRE) domain and a C-terminal catalytic domain. The RRE domain is essential for recognizing the substrate, as demonstrated by in vivo studies where its deletion abolishes product formation [19]. The catalytic mechanism of GrcB proceeds in two key steps. Initially, GrcB uses ATP and Mg^2+^ to adenylylate the C-terminal carboxylate of GrcA, forming a peptide-AMP intermediate. Subsequently, the enzyme enables the thiol group of Cys39 on GrcA to attack this intermediate, releasing AMP and forming a thioester bond that completes macrocyclization [19]. This process resembles the adenylyl transfer catalyzed by ThiF in thiamine biosynthesis, yet it differs in substrate specificity and final product [24]. Additionally, GrcB prefers substrates pre-modified by GrcC to incorporate L-allo-Thr, suggesting that GrcC’s epimerization precedes and enhances GrcB’s activity, highlighting the coordinated interplay among enzymes in the grc biosynthetic gene cluster [19].

The identification and characterization of GrcB as a ThiF-like enzyme within the grc biosynthetic gene cluster (BGC) underscore its unique role in catalyzing the formation of a thiolactone macrocycle. This discovery not only expands the known reaction capabilities of the ThiF/E1 enzyme superfamily but also enhances our understanding of the enzymology driving RiPP biosynthesis [19]. The biological significance of this thiolactone macrocycle in RiPP biosynthesis, however, remains to be fully explored. Unlike the five-amino-acid thiolactones observed in *Staphylococcus aureus* autoinducing peptides (AIPs), GrcB facilitates an intramolecular reaction between the side-chain of an internal glutamate and a C-terminal cysteine, operating in a reverse orientation compared to conventional AIPs [85]. Previously, thiolactone macrocycles in RiPPs were primarily linked to enzymes such as AgrD and AgrB, which are involved in quorum sensing [85]. The GRC-type RiPPs may function as quorum-sensing effector molecules, although this remains to be validated through further experimental studies. Thus, the discovery of a ThiF-like enzyme mediating this process broadens our understanding of RiPP cyclization mechanisms and opens new avenues for exploring their functional roles.

### 4.2. Genome Mining for GRC-Type RiPPs

In a previous study, *Seyedsayamdost* et al. used a bioinformatics strategy to identify the GRC subfamily by searching for gene clusters that contain both quorum sensing regulatory systems and Radical SAM proteins [17,19]. This subfamily is mainly found in *S. pneumoniae*, a pathogen that causes bacterial pneumonia. Interestingly, the infrequent occurrence of the ThiF protein in these gene clusters suggests that alternative ThiF-like proteins—those that are structurally or functionally similar yet distinct—may be involved in the biosynthesis of currently uncharacterized RiPPs [17,19].

**Figure 8:**
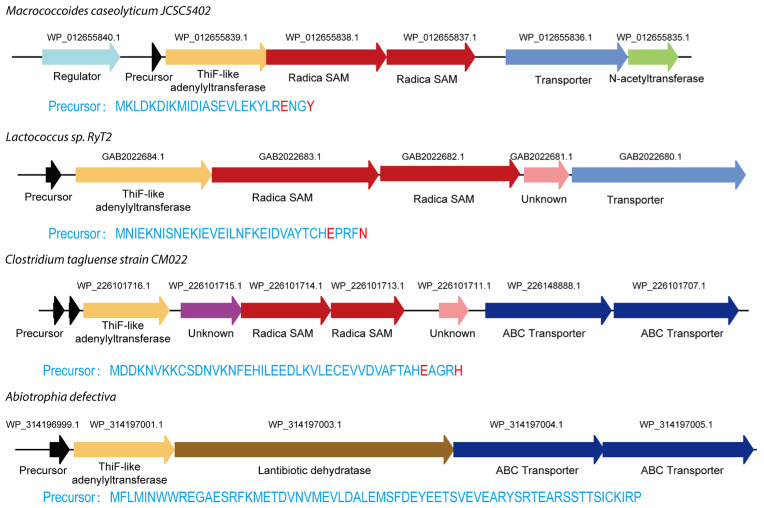
Genome mining for biosynthetic gene clusters of GRC-type RiPPs found across bacterial genomes.

To further explore the distribution of GRC-related gene clusters, a BLAST search of the NCBI database using GrcB as a query sequence was conducted. Indeed, bioinformatic analysis revealed several GRC-like clusters in bacterial species beyond *S. pneumoniae* (Figure 8). For example, related clusters occur in *Lactococcus* sp. RyT2 and *Macrococcoides caseolyticum* JCSC5402. Notably, the cluster in *M. caseolyticum* JCSC5402 includes an additional N-acetyltransferase, suggesting that its final product may undergo more complex chemical modifications. Moreover, a gene cluster in *Clostridium tagluense* strain CM022 contains proteins with unknown domains, indicating that its metabolic products could exhibit even greater structural complexity. Another interesting aspect concerns the precursor. While the GRC system forms a thiolactone macrocycle by cross-linking the C-terminal glutamate (Glu^35^) to cysteine (Cys^39^), the newly identified precursors—with different C-terminal amino acids—point to the existence of novel macrocyclic peptides.

Of particular note, a gene cluster in *Abiotrophia defectiva* that contains a ThiF-like adenylyltransferase alongside a lanthipeptide dehydratase was identified. This finding suggests that ThiF proteins may have a broader catalytic repertoire than previously recognized. Further functional characterization of these ThiF homologs could reveal novel ThiF-mediated biochemical transformations, shedding new light on the diversity of RiPP biosynthetic pathways and their post-translational modifications.

## 5. TLATs in Enterofaecin-Type RiPPs Biosynthesis

### 5.1. Biosynthesis of Enterofaecin-Type RiPPs

*Lactobacilli* are Gram-positive bacteria that include both pathogenic and probiotic strains. The probiotic strains are widely used in industrial fermentations. Despite their small genomes, these bacteria have a wealth of untapped biosynthetic potential. Recently, using the TLAT (MccB) from the *E. coli* microcin C system as a query, we conducted a PSI-BLAST search for homologous sequences in *Lactobacilli* genomes and analyzed their distribution via sequence similarity networks [22]. The results reveal that TLATs are ubiquitously present in *Lactobacilli* and are closely associated with multiple novel RiPPs biosynthetic gene clusters. In particular, these gene clusters are classified into several evolutionary clades within genera such as *Enterococcus* and *Streptococcus*, with the WWIII-type gene cluster being one of the most representative [22].

**Figure 9:**
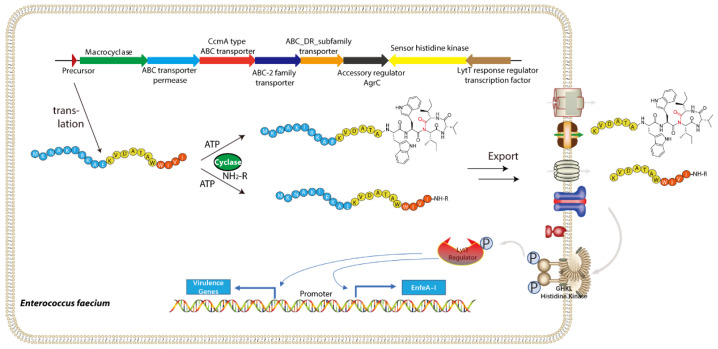
Proposed schematic of enterofaecin biosynthesis. Precursor peptide EnfA is first either cyclized or amidated [22].

In detailed study of *Enterococcus faecalis* FDAARGOS_397, a unique gene cluster that encodes a 21-residue precursor peptide named EnfA was identified, which features the characteristic WWIVI motif [22]. This cluster also includes the TLAT enzyme EnfB, four transporter proteins, and several regulatory enzymes and is named the enterofaecin biosynthetic pathway (Figure 9) [22]. Further, in vitro studies demonstrate EnfB’s critical role in facilitating the intramolecular cyclization of the precursor peptide, providing new insight into how to expand the chemical diversity of natural products [22].

Enterofaecin biosynthesis begins with the linear precursor peptide EnfA, which has an N-terminal leader sequence and a C-terminal core sequence. In vitro assays show that EnfB activates the C-terminal carboxylate of EnfA through an ATP/Mg^2+^-dependent mechanism, producing a reactive acyl-adenylate intermediate [22]. Then, the activated C-terminal acyl-AMP anhydride is attacked by the backbone amide nitrogen of Ile19 in EnfA, leading to the formation of a macrocyclic imide structure [22]. Multiple mass spectrometry and NMR analysis confirm this cyclization, identifying the reaction site as the conserved WIVI sequence at the C-terminus. Additionally, under specific conditions, EnfB can catalyze an amidation of the activated C-terminus using available amines. This expands its catalytic capabilities and may influence the bioactivity and transport of the final product in vivo [22].

Structurally and mechanistically, EnfB resembles classical TLATs, such as MccB, yet displays several distinctions. Kinetic monitoring of the in vitro reaction, together with structural models generated by AlphaFold, revealed that EnfB consists of two main domains: an N-terminal RiPP precursor recognition element (RRE) that binds the leader peptide, and a C-terminal adenylyltransferase domain that catalyzes ATP-dependent substrate activation [22]. Comparative analysis with MccB showed that conserved residues Arg167 and Lys180 are essential for ATP binding, while Asp225 coordinates Mg^2+^ to stabilize ATP’s negative charge—a role confirmed by site-directed mutagenesis, since substituting Asp225 with alanine abolished enzymatic activity. Unlike MccB, which uses two ATP molecules to form an N-P bond, EnfB carries out both cyclization and amidation using a single ATP. Its downstream pathway then diverges depending on the availability of exogenous primary amines, producing either a macrocyclic imide or an amide-modified product [22].

Biochemical studies show that EnfB relies heavily on the precursor peptide’s N-terminal leader sequence, with the conserved (V/I)RKA motif being particularly important [22]. Additionally, Trp17 and Trp18 in the core sequence are crucial for substrate binding and catalytic activity. Interestingly, EnfB tolerates some variation near the C-terminus, as certain modified precursors still undergo reaction [22]. This flexibility suggests that EnfB might accept a range of substrates, potentially leading to a variety of modified peptides.

This example also enhances our understanding of RiPP biosynthetic strategies and offers a powerful tool for creating new cyclic scaffolds. Unlike traditional NRPS systems, EnfB performs activation, cyclization, and C-terminal amidation in a single ATP-dependent step [22]. Moreover, the gene cluster’s inclusion of multiple ATP-binding cassette transporter genes and quorum sensing-related regulatory factors suggests that enterofaecin and similar natural products may function as quorum-sensing effector molecules and contribute to bacterial virulence and intercellular communication [22]. Exploring the biological roles of EnfB and its products could uncover new targets for antimicrobial therapies and the development of innovative signaling molecules [22].

### 5.2. Genome Mining for Enterofaecin-Type RiPPs

**Figure 10:**
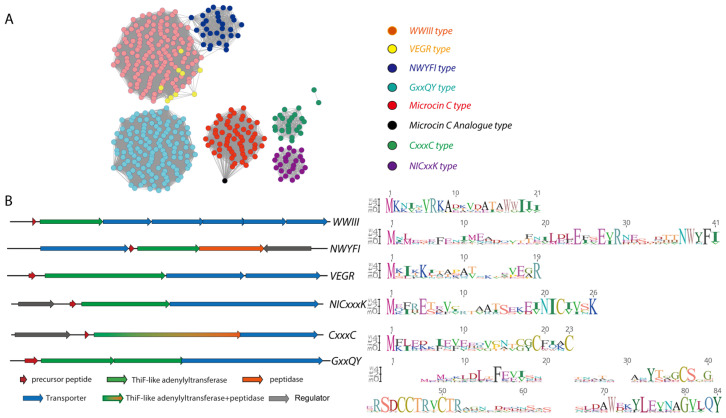
ThiF-RiPP Network in Lactobacillales. (**A**) sequence similarity network of ThiF-RiPP gene clusters from 8 groups (edge % identity of 20) based on the sequence of the ThiF genes; (**B**) Representative biosynthetic gene cluster for each of the sub-families in panel A. Precursor peptide logo plots for ThiF-RiPP subfamilies are shown on the right [22].

Genome mining shows that TLAT biosynthetic pathways are common in lactobacilli. Using the MccB sequence from the *E. coli* microcin C system (NCBI AAY68495.1) as a query, PSI-BLAST searches on *Enterococcus* and *Streptococcus* uncovered many similar sequences, which were grouped into nine distinct clusters in *Enterococcus* and eleven in *Streptococcus* using sequence similarity networks (Figure 10) [22]. A closer look at the TLAT genes and their surrounding regions revealed connections to transporter proteins, precursor peptides, and various modifying enzymes, such as decarboxylases, methyltransferases, N-acetyltransferases, nucleotide transferases, PLP-dependent enzymes, and radical SAM enzymes [22]. These pathways often appear near transposons, hinting that they may spread between bacteria through horizontal gene transfer. Interestingly, species like *Streptococcus pneumoniae* and *Enterococcus faecalis* can have up to seven unique TLAT-RiPP pathways, emphasizing the key role of TLAT-RiPP metabolites in Lactobacilli.

For example, the NWYFI group, exclusive to Lactobacilli and mainly *streptococci* with 31 instances, features longer precursors and specific enzymes, including a peptidase, a transporter, and a regulator. Collectively, these findings demonstrate that nature widely employs TLATs to diversify RiPP functionalities, revealing a rich and complex landscape of potential natural products with untapped biological activities [22].

## 6. Conclusions

RiPPs have emerged as a class of microbial natural products that have attracted considerable attention in recent years [10]. With rapid advances in genomic mining and bioinformatics, even bacterial taxa traditionally regarded as poor sources of natural products—such as streptococci and enterococci—are now being recognized for their great potential [17,21]. Their genomes harbor abundant biosynthetic gene clusters for RiPPs, suggesting that these microbes employ peptide-based natural products to mediate functions like antibacterial activity, cell-to-cell communication, biofilm formation, and metal acquisition [17]. In complex and competitive ecological niches, particularly within the gut microbiomes of mammals and humans, symbiotic bacteria have evolved a diverse arsenal of effector molecules to secure a competitive edge for resource and niche acquisition [86]. Although the biological functions of many RiPPs remain poorly understood, their roles in bacterial communication and physiological regulation may be underestimated, as their expression is tightly governed by quorum sensing systems [17,87]. Future interdisciplinary studies will be essential for fully elucidating their biological significance.

ThiF-like adenylyltransferases represent a class of structurally diverse and functionally versatile enzymes, playing critical roles in thiamine (vitamin B1) biosynthesis, ubiquitin-like protein activation, maturation of natural products, and bacterial toxin–antitoxin systems [10,11]. Recent advances have significantly enhanced our understanding of the role of ThiF-like enzymes in RiPP modification. Studies on the metabolic pathways for Microcin C, Pantocin A, GRC-type RiPPs, and Enterofaecin have revealed that these enzymes share a conserved NTP-dependent activation mechanism. However, their catalytic processes and the resulting products are remarkably diverse, demonstrating an exceptional substrate adaptability.

Looking forward, many exciting research avenues remain unexplored. Genomic mining combined with sequence similarity network analyses have already identified a substantial number of ThiF-like adenylyltransferases implicated in novel RiPP pathways that warrant further investigation. With ongoing advancements in genome mining, structural biology, and high-throughput biochemical screening, it is anticipated that additional uncharacterized ThiF-like enzymes and their corresponding natural products will be discovered. Moreover, given their unique ability to activate peptide substrates, these enzymes hold tremendous potential for applications in drug discovery and synthetic biology as biocatalysts. In-depth exploration of these biosynthetic pathways may ultimately provide entirely new strategies for the development of innovative natural drugs and biocatalysts.

## Data Availability

The data presented in this study are available on request from the corresponding author.
